# Supplementary material for: Fasting Normoglycemia after Intravenous Thrombolysis Predicts Favorable Long-Term Outcome in Non-Diabetic Patients with Acute Ischemic Stroke
Source: J Clin Med. 2021 Jul 6;10(14):3005. doi: 10.3390/jcm10143005 (PMC8306150; doi:10.3390/jcm10143005)
Supplement: Supplementary file 1 [file jcm-10-03005-s001.zip › jcm-1206318-supplementary.pdf]

Supplemental Table S1. Baseline characteristics of patients according to modified Rankin scale (mRS).

	mRS 0-2<br>n=231	mRS 3-6<br>n=121	P - value	mRS 0-1<br>n=206	mRS 2-6<br>n=146	P - value
Age (years)	70 (59-79)	78 (69-83)	<0.001	69.5 (59-78)	77 (68-83)	<0.001
Sex (female)	101 (43.7)	72 (59.5)	0.005	90 (43.7)	83 (56.9)	0.015
BMI (kg/m^2)	26.6 (24.1-29.3)	26.9 (24.5-30.5)	0.312	26.4 (24.1-29.3)	27.2 (24.3-30.5)	0.162
Hypertension, n (%)	179 (77.5)	115 (95.0)	<0.001	157 (76.2)	137 (93.8)	<0.001
Ischemic heart disease, n (%)	49 (21.1)	33 (27.2)	0.201	45 (21.8)	37 (25.3)	0.445
Atrial fibrillation, n (%)	58 (25.1)	45 (37.2)	0.179	52 (25.2)	51 (34.9)	0.049
Hipercholesterolemia, n (%)	79 (34.2)	31 (25.6)	0.099	69 (33.5)	41 (28.1)	0.280
Smoking, n (%)	40 (17.3)	14 (11.6)	0.155	35 (17.0)	19 (13.0)	0.307
Previous stroke, n (%)	35 (15.2)	28 (23.1)	0.063	31 (15.1)	32 (21.9)	0.098
Stroke etiology, n (%)						
- large-vessel disease	33 (14.3)	14 (11.6)	0.301	26 (12.6)	21 (14.4)	0.411
- small-vessel disease	3 (1.3)	0 (0.0)		2 (1.0)	1 (0.7)	
- cardioembolic	69 (29.9)	48 (39.7)		61 (29.6)	56 (38.4)	
- other etiology	12 (5.2)	5 (4.1)		11 (5.3)	6 (4.1)	
- undetermined etiology	114 (49.3)	54 (44.6)		106 (51.5)	(62 (42.5)	
Mechanical thrombectomy, n (%)	62 (26.8)	43 (35.5)	0.090	50 (24.3)	55 (37.7)	0.007
Time from stroke onset to thrombolysis (min)	130 (95-183)	135 (95-180)	0.977	131 (95-185)	135 (93-180)	0.775
NIHSS score on admission	9.7±6.0	16.3±6.0	<0.001	9.1±5.7	16.0±6.0	<0.001
NIHSS score after r-tPA	4.5±4.3	16.0±7.6	<0.001	3.9±3.8	14.8±7.6	<0.001
Post-MT hemorrhagic brain complications, n (%)						
- no complication	208 (90.0)	71 (58.7)	<0.001	187 (90.8)	92 (63.0)	<0.001
- HI type 1	10 (4.3)	14 (11.6)		9 (4.4)	15 (10.3)	
- HI type 2	8 (3.5)	15 (12.4)		8 (3.9)	15 (10.3)	
- PH type 1	5 (2.2)	9 (7.4)		2 (1.0)	12 (8.2)	
- PH type 2	0 (0.0)	12 (9.9)		0 (0.0)	12 (8.2)	
Maximal SBP within 24 hours after r-tPA (mmHg)	145 (126-160)	146 (137-165)	0.048	145 (126-159)	146.5 (136-166)	0.019
Maximal DBP within 24 hours after r-tPA (mmHg)	80 (70-85)	80 (72-90)	0.107	80 (71-85)	80 (70-90)	0.278
Fasting glucose (mmol/L)	6.4 (5.5-7.9)	6.9 (6.1-8.6)	0.004	6.3 (5.5-7.7)	7.0 (6.1-8.8)	<0.001
Fasting glucose < 5.5 mmol/L	56 (24.2)	8 (6.6)	<0.001	53 (25.7)	11 (7.5)	0.001
Death (mRS=6)	0 (0.0)	54 (44.6)	<0.001	0 (0.0)	54 (37.0)	<0.001

Values are presented as n (%), mean ± standard deviation, or median and interquartile range. Abbreviations: BMI – body mass index, DBP – diastolic blood pressure, HI – hemorrhagic infarction, mRS – modified Rankin scale, MT – mechanical thrombectomy, NIHSS – National Institutes of Health Stroke Scale, PH – parenchymal hematoma, r-tPA – recombinant tissue plasminogen activator, SBP – systolic blood pressure.

Supplemental Table S2. Baseline characteristics of patients treated only with intravenous thrombolysis (without mechanical thrombectomy) according to modified Rankin scale (mRS).

	mRS 0-2<br>n=168	mRS 3-6<br>n=79	P - value	mRS 0-1<br>n=156	mRS 2-6<br>n=91	P - value
Age (years)	71 (62-80)	80 (72-84)	<0.001	71 (62-80)	79 (71-84)	<0.001
Sex (female)	78 (46.4)	45 (57.0)	0.122	73 (46.8)	50 (55.0)	0.216
BMI (kg/m^2)	26.5 (23.8-29.4)	26.5 (24.2-30.1)	0.562	26.4 (23.8-29.3)	26.4 (24.2-30.9)	0.447
Hypertension, n (%)	131 (78.0)	76 (96.2)	<0.001	120 (76.9)	87 (95.6)	<0.001
Ischemic heart disease, n (%)	37 (22.0)	25 (31.7)	0.104	35 (22.4)	27 (29.7)	0.205
Atrial fibrillation, n (%)	37 (22.0)	33 (41.8)	0.001	36 (23.1)	34 (37.4)	0.017
Hipercholesterolemia, n (%)	65 (38.7)	25 (31.7)	0.283	58 (37.2)	32 (35.2)	0.751
Smoking, n (%)	28 (16.7)	8 (10.1)	0.174	26 (16.7)	10 (11.0)	0.222
Previous stroke, n (%)	31 (18.5)	22 (27.9)	0.093	29 (18.6)	24 (26.4)	0.151
Stroke etiology, n (%)						
- large-vessel disease	26 (15.5)	7 (8.9)	0.071	21 (13.5)	12 (13.2)	0.219
- small-vessel disease	2 (1.2)	1 (1.3)		2 (1.3)	1 (1.1)	
- cardioembolic	44 (26.2)	35 (44.3)		42 (26.9)	37 (40.7)	
- other etiology	5 (3.0)	2 (2.5)		4 (2.6)	3 (3.3)	
- undetermined etiology	91 (54.2)	34 (43.0)		87 (55.8)	38 (41.8)	
Time from stroke onset to thrombolysis (min)	145 (100-200)	148 (95-195)	0.784	142 (100-191)	150 (95-205)	0.821
NIHSS score on admission	7.8±5.1	15.3±5.7	<0.001	7.5±4.8	14.8±5.9	<0.001
NIHSS score after r-tPA	3.6±3.6	14.8±6.6	<0.001	3.2±3.3	13.9±6.8	<0.001
Post-MT hemorrhagic brain complications, n (%)						
- no complication	158 (94.1)	56 (70.9)	<0.001	146 (93.6)	68 (74.7)	<0.001
- HI type 1	5 (3.0)	8 (10.1)		5 (3.2)	8 (8.8)	
- HI type 2	4 (2.4)	4 (5.1)		4 (2.6)	4 (4.4)	
- PH type 1	1 (0.6)	5 (6.3)		1 (0.6)	5 (5.5)	

- PH type 2	0 (0.0)	6 (7.6)		0 (0.0)	6 (6.6)	
Maximal SBP within 24 hours after r-tPA (mmHg)	145 (128-160)	147 (130-166)	0.247	145 (128-160)	148 (130-166)	0.152
Maximal DBP within 24 hours after r-tPA (mmHg)	80 (70-85)	80 (70-90)	0.320	80 (72-85)	80 (70-90)	0.671
Fasting glucose (mmol/L)	6.4 (5.5-7.8)	7.1 (6.1-8.9)	0.002	6.3 (5.5-7.7)	7.1 (6.2-9.1)	<0.001
Fasting glucose < 5.5 mmol/L	16 (21.4)	4 (5.1)	<0.001	35 (22.4)	5 (5.5)	0.001
Death (mRS=6)	0 (0.0)	35 (44.3)	<0.001	0 (0.0)	35 (38.5)	<0.001

For abbreviations, see Supplemental Table 1.

Supplemental Table S3. Multivariable logistic regression model for favorable (mRS 0-2) and excellent (mRS 0-1) 3-month clinical outcome in patients treated only with intravenous thrombolysis (without mechanical thrombectomy).

Favorable outcome (mRS 0-2)						
	Univariable			Multivariable		
90-day favorable clinical outcome, diabetic + non-diabetic patients	HR	95% CI	P-value	HR	95% CI	P-value
Age (per 1 year)	0.93	0.91-0.96	<0.001	0.94	0.90-0.98	0.005
Sex (female)	0.66	0.38 -1.12	0.123	-	-	-
BMI (per 1 unit)	0.97	0.91-1.03	0.299	-	-	-
Atrial fibrillation	0.39	0.22-0.70	0.002	-	-	
Hypertension	0.14	0.04-0.47	0.001			
NIHSS score after r-tPA (per 1 point)	0.71	0.65-0.77	<0.001	0.71	0.65-0.77	<0.001
Hemorrhagic brain complications (ECASS 1-3)	0.15	0.07-0.34	<0.001	-	-	-
Fasting glucose <5.5 mmol/l	5.11	1.75-14.93	0.003	3.87	0.89-16.77	0.071
90-day favorable clinical outcome, diabetic patients						
Age (per 1 year)	0.93	0.88-0.99	0.023	0.83	0.73-0.95	0.006
Sex (female)	1.45	0.57-3.97	0.421	-	-	-
BMI (per 1 unit)	0.88	0.78-0.98	0.025	0.77	0.61-0.95	0.017
Atrial fibrillation	0.29	0.10-0.80	0.017	-	-	-
Previous stroke	0.30	0.10-0.90	0.032	-	-	-
NIHSS score after r-tPA (per 1 point)	0.70	0.60-0.82	<0.001	0.61	0.48-0.79	<0.001
Hemorrhagic brain complications (ECASS 1-3)	0.40	0.08-1.96	0.260	-	-	-
Creatinine (per 1 μmol/l)	0.98	0.97-0.99	0.023	-	-	-
Fasting glucose <5.5 mmol/l	1.45	0.34-6.18	0.615	-	-	-
90-day favorable clinical outcome, non-diabetic patients						

Age (per 1 year)	0.94	0.91-0.97	<0.001	0.92	0.87-0.98	0.005
Sex (female)	0.45	0.23-0.87	0.017	-	-	-
BMI (per 1 unit)	1.03	0.95-1.12	0.507	-	-	-
Atrial fibrillation	0.48	0.23-0.96	0.039	-	-	-
Hypertension	0.17	0.05-0.57	0.004	-	-	-
Maximal SBP within 24 hours after r-tPA (per 1 mmHg)	0.99	0.98-0.99	0.035	1.04	1.01-1.07	0.004
NIHSS score after r-tPA (per 1 point)	0.70	0.63-0.77	<0.001	0.65	0.56-0.75	<0.001
Hemorrhagic brain complications (ECASS 1-3)	0.11	0.04-0.28	<0.001	-	-	-
Fasting glucose <5.5 mmol/l	15.87	2.10-119.9	0.007	28.61	1.19-436.2	0.036

Excellent outcome (mRS 0-1)						
	Univariable			Multivariable		
90-day excellent clinical outcome, diabetic + non-diabetic patients	HR	95% CI	p-value	HR	95% CI	p-value
Age (per 1 year)	0.94	0.92-0.97	<0.001	0.95	0.92-0.98	0.003
Sex (female)	0.72	0.43-1.21	0.217	-	-	-
BMI (per 1 unit)	0.97	0.91-1.03	0.267	-	-	-
Hypertension	0.15	0.05-0.45	<0.001	-	-	-
Atrial fibrillation	0.50	0.29-0.89	0.017	-	-	-
NIHSS score after r-tPA (per 1 point)	0.70	0.64-0.76	<0.001	0.70	0.63-0.76	<0.001
Hemorrhagic brain complications (ECASS 1-3)	0.20	0.09-0.45	<0.001	-	-	-
Fasting glucose <5.5 mmol/l	4.98	1.87-13.21	0.001	4.79	1.17-19.20	0.027
90-day excellent clinical outcome, diabetic patients						
Age (per 1 year)	0.95	0.91-1.01	0.078	-	-	-
Sex (female)	1.81	0.70-4.68	0.223	-	-	-
BMI (per 1 unit)	0.87	0.77-0.98	0.017	0.77	0.61-0.97	0.025
Atrial fibrillation	0.41	0.15-1.10	0.077	-	-	-
NIHSS score after r-tPA (per 1 point)	0.63	0.51-0.78	<0.001	0.55	0.41-0.75	<0.001
Hemorrhagic brain complications (ECASS 1-3)	0.60	0.12-2.91	0.525	-	-	-
Creatinine (per 1 μmol/l)	0.98	0.96-0.99	0.010	0.96	0.93-0.99	0.019
Fasting glucose <5.5 mmol/l	2.18	0.51-9.25	0.289	-	-	-
90-day excellent clinical outcome, non-diabetic patients						
Age (per 1 year)	0.94	0.91-0.97	<0.001	0.96	0.92-0.99	0.034
Sex (female)	0.47	0.25-0.89	0.020	-	-	-

BMI (per 1 unit)	1.03	0.96-1.20	0.408	-	-	-
Atrial fibrillation	0.60	0.30-1.20	0.145	-	-	-
Hypertension	0.20	0.07-0.58	0.003	-	-	-
Maximal SBP within 24 hours after r-tPA (per 1 mmHg)	0.98	0.97-0.99	0.003	-	-	-
NIHSS score after r-tPA (per 1 point)	0.71	0.64-0.78	<0.001	0.71	0.65-0.79	<0.001
Hemorrhagic brain complications (ECASS 1-3)	0.13	0.05-0.34	<0.001	-	-	-
Fasting glucose <5.5 mmol/l	8.87	2.03-38.66	0.004	6.47	0.87-48.15	0.068

For abbreviations, see Supplemental Table 1; ECASS – The European Cooperative Acute Stroke Study.

Supplemental Table S4. Multivariable logistic regression model for the risk of death in patients treated only with intravenous thrombolysis (without mechanical thrombectomy).

	Univariable			Multivariable		
90-day risk of death, diabetic + non-diabetic patients	HR	95% CI	p-value	HR	95% CI	p-value
Age (per 1 year)	1.07	1.03-1.12	<0.001	1.07	1.02-1.11	0.009
Sex (female)	1.41	0.69-2.90	0.350	-	-	-
BMI (per 1 unit)	0.99	0.91-1.08	0.784	-	-	-
Atrial fibrillation	2.83	1.36-5.89	0.005	-	-	-
Hypertension	3.60	0.82-15.70	0.087	-	-	-
Maximal SBP within 24 hours after r-tPA (per 1 mmHg)	1.01	1.00-1.03	0.065	-	-	-
NIHSS score after r-tPA (per 1 point)	1.25	1.17-1.33	<0.001	1.24	1.16-1.32	<0.001
Hemorrhagic brain complications (ECASS 1-3)	6.77	2.97-15.44	<0.001	-	-	-
Fasting glucose <5.5 mmol/l	1.13	0.02-0.98	0.048	-	-	-

For abbreviations, see Supplemental Table 1 and 2.

Supplemental Figure S1

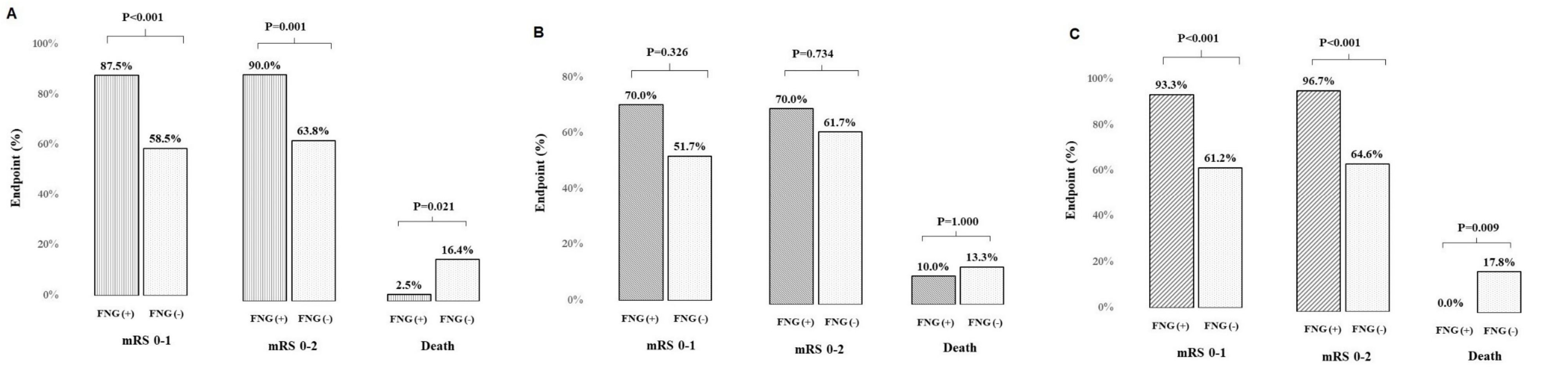

Patients treated only with intravenous thrombolysis (without mechanical thrombectomy) with favorable (mRS 0-2) or excellent (mRS 0-1) outcome and those who died according to the presence of fasting normoglycemia in the whole group (A), in the subgroup with (B), and without diabetes mellitus (C).
